# Mutations in foregut SOX2^+^ cells induce efficient proliferation via CXCR2 pathway

**DOI:** 10.1007/s13238-019-0630-3

**Published:** 2019-04-30

**Authors:** Tomoaki Hishida, Eric Vazquez-Ferrer, Yuriko Hishida-Nozaki, Ignacio Sancho-Martinez, Yuta Takahashi, Fumiyuki Hatanaka, Jun Wu, Alejandro Ocampo, Pradeep Reddy, Min-Zu Wu, Laurie Gerken, Reuben J. Shaw, Concepcion Rodriguez Esteban, Christopher Benner, Hiroshi Nakagawa, Pedro Guillen Garcia, Estrella Nuñez Delicado, Antoni Castells, Josep M. Campistol, Guang-Hui Liu, Juan Carlos Izpisua Belmonte

**Affiliations:** 1 Gene Expression Laboratory, Salk Institute for Biological Studies, 10010 North Torrey Pines Road, La Jolla, CA 92037 USA; 2 Universidad Catolica, San Antonio de Murcia, Campus de los Jeronimos 135, Guadalupe, 30107 Spain; 3 Molecular and Cell Biology Laboratory, Dulbecco Center for Cancer Research, Salk Institute for Biological Studies, 10010 North Torrey Pines Road, La Jolla, CA 92037 USA; 4 Howard Hughes Medical Institute, Dulbecco Center for Cancer Research, Salk Institute for Biological Studies, 10010 North Torrey Pines Road, La Jolla, CA 92037 USA; 5 Integrative Genomics Core, Salk Institute for Biological Studies, 10010 North Torrey Pines Road, La Jolla, CA 92037 USA; 6 Division of Gastroenterology, Department of Medicine, Perelman School of Medicine, University of Pennsylvania, Philadelphia, PA 19104 USA; 7 Abramson Cancer Center, University of Pennsylvania, Philadelphia, PA 19104 USA; 8 Department of Traumatology and Research Unit, Clinica CEMTRO, Av. Ventisquero de la Condesa, 42, Madrid, 28035 Spain; 9 Gastroenterology Department, Hospital Clinic, University of Barcelona, IDIBAPS, CIBEREHD, Barcelona, 08036 Spain; 10 Advanced Innovation Center for Human Brain Protection, National Clinical Research Center for Geriatric Disorders, Xuanwu Hospital Capital Medical University, Beijing, 100053 China; 11 National Laboratory of Biomacromolecules, CAS Center for Excellence in Biomacromolecules, Institute of Biophysics, Chinese Academy of Sciences, Beijing, 100101 China; 12 University of the Chinese Academy of Sciences, Beijing, 100049 China; 13 Insitute for Stem Cell and Regeneration, Chinese Academy of Sciences, Beijing, 100101 China; 14 Beijing Institute for Brain Disorder, Beijing, 100069 China

**Keywords:** Sox2, tumor, CXCR2, stratified epithelia

## Abstract

Identification of the precise molecular pathways involved in oncogene-induced transformation may help us gain a better understanding of tumor initiation and promotion. Here, we demonstrate that SOX2^+^ foregut epithelial cells are prone to oncogenic transformation upon mutagenic insults, such as *Kras*^*G12D*^ and *p53* deletion. GFP-based lineage-tracing experiments indicate that SOX2^+^ cells are the cells-of-origin of esophagus and stomach hyperplasia. Our observations indicate distinct roles for oncogenic KRAS mutation and P53 deletion. *p53* homozygous deletion is required for the acquisition of an invasive potential, and *Kras*^*G12D*^ expression, but not *p53* deletion, suffices for tumor formation. Global gene expression analysis reveals secreting factors upregulated in the hyperplasia induced by oncogenic KRAS and highlights a crucial role for the CXCR2 pathway in driving hyperplasia. Collectively, the array of genetic models presented here demonstrate that stratified epithelial cells are susceptible to oncogenic insults, which may lead to a better understanding of tumor initiation and aid in the design of new cancer therapeutics.

**Electronic supplementary material:**

The online version of this article (10.1007/s13238-019-0630-3) contains supplementary material, which is available to authorized users.

## Introduction

Cancer arises from a progressive accumulation of genetic mutations in proto-oncogenes and tumor suppressor genes (Visvader and Lindeman, 2012; Blanpain and Simons, 2013). For example, the oncogene *Kras* and the tumor suppressor gene *p53* are frequently mutated in a wide range of human cancers (Serrano et al. 1997; Kuilman et al., 2010) and are known to induce tumor initiation in a variety of mouse models (Jackson et al., 2001; Singh et al., 2010).

Abnormal proliferative signals of oncogenic insults including oncogenic KRAS are known to activate a senescent phenotype in cells, presumably designed to prevent the growth of oncogene-transformed cells and to preserve the tumor in a non-aggressive state (Collado and Serrano, 2006). Senescent cells, in turn, secrete large amounts of cytokines and chemokines in a phenomenon known as Senescence-Associated Secretory Phenotype (SASP). Among SASP-related factors, CXC chemokines that bind to CXC chemokine receptor 2 (CXCR2) have been shown to reinforce senescence, which results in growth arrest, further preventing tumor progression (Acosta et al., 2008). However, SASP components can also dangerously stimulate a malignant phenotype and have tumor-promoting responses. Some of the factors secreted by senescent cells such as GROα, CXCL-12 or IL-8 lead to activate proliferation in the surrounding epithelial cells (Krtolica et al., 2001; Coppé et al., 2008). Therefore, the effect of SASP on cell behavior is context-dependent.

Not only is the specific genetic mutation a determining factor for tumor initiation but the cell type from which the tumor originates is also important. Cellular populations that seem to have particularly high tumorigenic potential include adult stem cells (ASCs) and progenitor cells (PCs), which normally play crucial roles in tissue homeostasis and repair (Huels and Sansom, 2015; Sanchez-Danes et al., 2016; Zhu et al., 2016). These cells might be ideal candidates to serve as the cells-of-origin for cancers and as such ASCs/PCs have been intensively studied. However, it still remains to be fully understood which cell population is prone to oncogenic transformation and what kind of oncogenic insults induce tumor initiation from certain ASCs/PCs.

Here, we sought to identify proliferative ASCs/PCs that are the most susceptible to oncogenic mutations. By initially focusing on oncogenic *Kras*, together with the loss of *p53*, we found that foregut basal cells that express SOX2 efficiently proliferated to hyperplasia in response to oncogenic mutations. We also revealed distinct roles of oncogenic KRAS and P53 deletion in driving hyperplasia. Furthermore, oncogenic Kras elevated expression of SASP-related chemokines, which contributed to the oncogenic proliferation through a CXCR2-dependent signaling pathway. Taken together, these results suggest that SOX2^+^ epithelial basal cells in the esophagus and stomach are highly susceptible to oncogenic stimuli. Our findings may help elucidate early events in tumor formation and the cells-of-origin of tumors, which could in turn provide insights towards a better understanding of neoplasia.

## RESULTS

### Expressing oncogenic *Kras* and *p53* deletion in SOX2^+^ cells induces hyperplasia in the esophagus and forestomach

**Figure 1:**
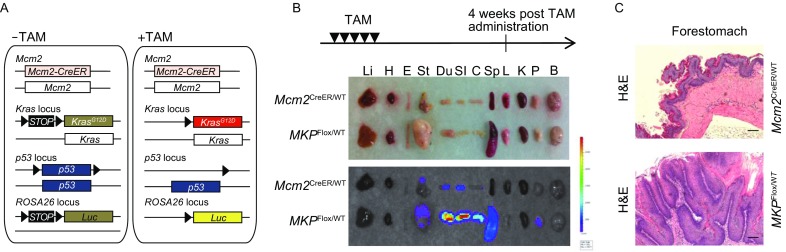
Susceptibility of proliferating cells to oncogenic stimuli. (A) Schematic representation of the genetic strategy for *Kras* and *p53* modifications in MCM2^+^ cells (MKP mouse model). (B) BLI analysis of *Mcm2*^CreER/WT^ or MKP^Flox/WT^ 4 weeks post tamoxifen (TAM) administration. Li: Liver; H: Heart; E: Esophagus; St: Stomach; Du: Duodenum; SI: Small intestine; C; Colon; Sp: Spleen; Lu: Lung; K: Kidney; P: Pancreas; B: Brain. (C) H&E on paraffin-embedded sections from *Mcm2*^CreER/WT^ mice and MKP mice. Scale bars, 100 μm

To determine which stem cell populations are the most vulnerable to oncogenic transformation, we expressed oncogenic *Kras* (G12D) and deleted one copy of the *p53* gene in dividing cells of the adult mouse. Oncogenic *Kras* and *p53* mutations were chosen because they are frequently observed in a wide range of human cancers (Serrano et al., 1997; Kuilman et al., 2010). We targeted proliferative cell populations using *Mcm2*-CreER knock-in mice (*Mcm2*^CreER/WT^), in which *CreER* expression is controlled by the *Mcm2* promoter. MCM2 is a component of the DNA replication licensing complex and localizes exclusively to proliferating cells. *Mcm2* expression is known to be downregulated when homozygous *Mcm2*-CreER mice (*Mcm2*^CreER/CreER^) are used, resulting in the loss of ASCs/PCs and the formation of cancer (likely because of genome instability) (Pruitt et al., 2007). *Mcm2*^CreER/WT^ mice were bred with mice carrying a *loxP*-STOP-*loxP* (LSL)-oncogenic *Kras* (G12D) (*Kras*^LSL-G12D/WT^) and *loxP*-p53-*loxP* mice (*p53*^Flox/Flox^) (Marino et al., 2000; Jackson et al., 2001). Upon genotyping, we verified and selected mice carrying the appropriate genetic modifications, namely *Mcm2*^CreER/WT^; *Kras*^G12D/WT^*; p53*^Flox/WT^ (hereafter referred to as MKP^Flox/WT^ mice). MKP^Flox/WT^ mice allow for the selective induction of *Kras*^*G12D*^ expression and the heterozygous deletion of *p53* in all dividing cells upon tamoxifen (TAM) administration. These mice also carried an LSL-*luciferase* (Luc) transgene in the *ROSA26* gene locus (*ROSA*^LSL-Luc/WT^) to allow for the visualization of *Cre*-expressing cells via bioluminescence imaging (BLI) (Fig. 1A). One month after TAM administration, we performed BLI of MKP^Flox/WT^ mice carrying *ROSA*^LSL-Luc/WT^ and noticed high levels of Luc expression, primarily in digestive tissues, including the small intestine (Fig. 1B). We also observed a prominent hyperplastic forestomach with abnormal proliferation of stratified epithelial layers (Fig. 1B and 1C). We repeated the experiments giving TAM intraperitoneally and the same phenotype was observed (data not shown). We then repeated this experiment using Cre lines restricted to stem cell populations, namely *Sox2*-CreER (SKP^Flox/WT^) and *Lgr5*-CreER (LKP^Flox/WT^), because SOX2 and LGR5 are known to mark ASC/PC populations in stratified epithelial squamous layers and in lower digestive tracts, respectively (Barker et al., 2007; Arnold et al., 2011). BLI revealed that Luc signals were specifically observed in the esophagus and stomach of SKP^Flox/WT^ mice, whereas LKP^Flox/WT^ mice exhibited strong Luc signals in the duodenum, small intestine, and colon (Fig. S1A), in agreement with previous reports (Feng et al., 2011; Snippert et al., 2014). We did not observe any hyperplasia in animals that lacked the CreER drivers (Fig. S1B).

**Figure 2:**
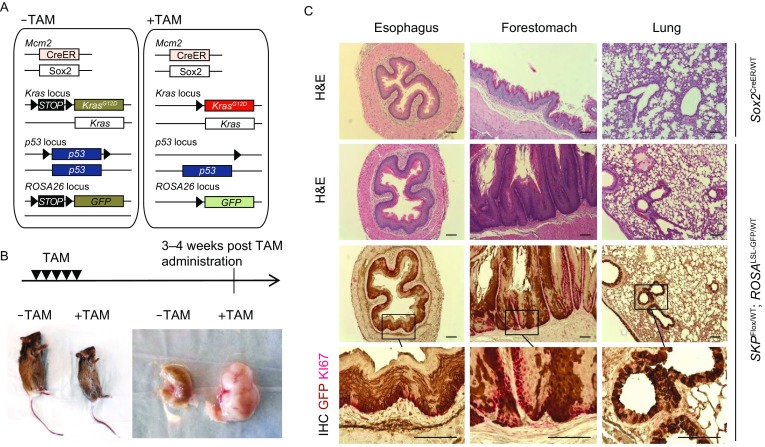
Cell susceptibility of foregut epithelial basal cells to oncogenic stimuli. (A) Schematic representation of SKP mouse carrying *ROSA*^LSL-GFP^ for lineage tracing purposes. (B) Stomachs collected from SKP^Flox/WT^ with or without treatment with TAM. (C) Lineage tracing experiment of SOX2^+^ cells in SKP^Flox/WT^ mice 3 weeks post TAM administration. Co-staining for GFP with KI67, a proliferative marker. Scale bars, 100 μm

Although SOX2 is expressed in a broad array of tissues, including lung, trachea, testis, tongue, pituitary gland, eye and brain (Que et al., 2009; Arnold et al., 2011), we did not see any hyperplasia in these tissues in the *Sox2*-CreER mice (Figs. 2A–C and S2, data not shown). Instead, we observed tissue-specific phenotypes, namely KRAS/P53-driven hyperplasia was generally restricted to the forestomach and esophagus with abnormalities in the glandular stomach. The hyperplasia was observed even in the older (3–4 month old) mice without any difference from the younger ones. We thus focused our attention on SOX2^+^ cells. SOX2 localizes to basal cells in the esophagus and forestomach, which are known to be progenitor cells with a high proliferative potential (Arnold et al., 2011; Doupe et al., 2012). To characterize hyperplasia in the esophagus and forestomach in more detail, we repeated the SOX2 experiment using a GFP marker (rather than the Luc marker) to allow for immunohistochemistry (IHC)-based lineage tracing of the SOX2^+^ ASCs/PCs in the esophagus, stomach, and lung (Fig. S3) after TAM administration. We confirmed the appearance of GFP^+^ cells 1 week after TAM administration in both the esophagus and forestomach (Fig. S4). IHC-based analysis of SKP^Flox/WT^ mice revealed GFP^+^ cells in the hyperplastic squamous region of the esophagus and forestomach and some of GFP^+^ cells were positive for KI67, a marker of proliferation (Fig. 2C). Analysis of the abnormalities found in the glandular stomach of SKP^Flox/WT^ mice revealed the presence of high amounts of mucosa, as assessed by Periodic acid-Schiff (PAS) staining (Fig. S5A). The alterations observed in the glandular region, however, were not directly linked to SOX2^+^ cells because we did not detect a clear increase in the GFP^+^ population with and without induction of oncogenic activity or a change in the expression pattern of differentiation markers of the glandular stomach proton-pump and gastrin (Fig. S5B). We next asked if oncogenic insults affected the differentiation potential of SOX2^+^ cells. These GFP^+^ cells were heterogeneous, with subpopulations expressing markers of undifferentiated (P63) or differentiated (CK13 and LORICRIN) cell types (Fig. S6), suggesting that the KRAS/P53 oncogenic stimulus does not affect the ability of these cells to differentiate, in contrast to what has been observed following *Sox2* overexpression (Liu et al., 2013). Previous reports showed that *Kras*^*G12D*^ does not seem to be commonly mutated in human esophageal squamous cell carcinoma (ESCC) (Shigaki et al., 2013), although related pathways are often activated (Lin et al., 2014) and this mutation is also observed in the Chinese population (Liu et al., 2011). Therefore, we next examined the effect of *PIK3CA* (H0147R), which is a mutation associated with ESCC (Lin et al., 2014; Song et al., 2014). Hyperplasia was also observed in the esophagus and forestomach when oncogenic *PIK3CA* was expressed together with heterozygous *p53* deletion (Fig. S7). Together, these results indicate that SOX2^+^ cells can be the cells-of-origin of forestomach and esophagus hyperplasia and suggest that SOX2^+^ basal cells in the esophagus and forestomach seem more susceptible to oncogenic stimuli than SOX2^+^ cells from other tissues in the body, implying tissue-specific vulnerabilities upon oncogenic insults.

### Differential impacts of oncogenic KRAS and P53 deletion on gene expression signature

**Figure 3:**
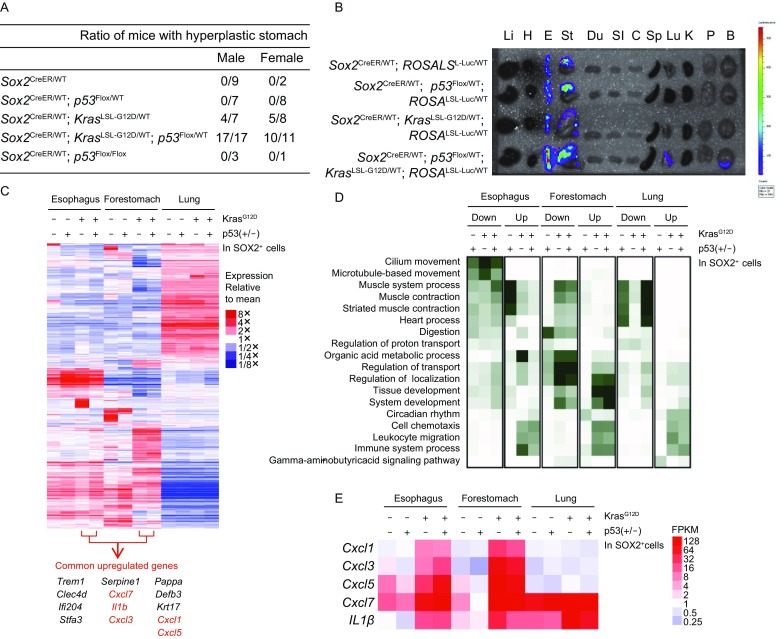
Distinct impacts of oncogenic KRAS and P53 modification. (A) Summary of the incidence of hyperplastic stomach. (B) *Ex vivo* BLI analysis of tissues from the indicated mice. Li: Liver; H: Heart; E: Esophagus; St: Stomach; Du: Duodenum; SI: Small intestine; C; Colon; Sp: Spleen; Lu: Lung; K: Kidney; P: Pancreas; B: Brain. (C) Heat map showing clustered gene expression profiles in indicated conditions using the results from RNA-Seq. Common genes that are upregulated by oncogenic Kras are extracted between the esophagus and stomach. (D) Gene Ontology enrichment for biological processes in genes significantly regulated by *Kras* and *p53* disruption in each tissue (FDR < 5%, fold-change > 2). (E) Heat map of common upregulated chemokine genes in Fig. 3C

To ascertain whether oncogenic Kras, heterozygous loss of p53, or both were responsible for induced hyperplasia in this context, we analyzed transgenic mice in which *Kras* and/or *p53* were manipulated using different combinations in SOX2^+^ cells. Upon TAM administration, stomach hyperplasia was only observed in animals that expressed mutant *Kras*, indicating that *Kras*^*G12D*^ expression, but not *p53* heterozygous deletion, was sufficient to induce the hyperplastic phenotype. Notably, hyperplasia was observed in almost all SKP^Flox/WT^ mice whereas lower rates were observed in mice carrying only mutant *Kras* (Fig. 3A), suggesting that deletion of one copy of *p53* accelerates tumorigenic proliferation by expanding SOX2^+^ cells, as supported by our BLI measurements (Fig. 3B) and IHC observations (Fig. S8). To characterize the molecular events that contribute to abnormal proliferation in the presence of oncogenic Kras, we next performed RNA-Sequencing (RNA-Seq) analysis using samples from the forestomach, esophagus, and lungs of *Sox2-*CreER mice with/without *Kras*^*G12D*^ and with/without one copy of the *p53* gene (see Fig. 3C). Clustering analysis revealed that gene expression signatures of esophagus and stomach tissue were altered by *Kras*^*G12D*^ expression with or without heterozygous *p53* deletion. In contrast, these genetic manipulations did not affect gene expression signatures in the lung, where proliferation was not observed. Gene ontology enrichment analysis further indicated a distinct impact of oncogenic KRAS versus P53 deletion (Fig. 3D). Because *Kras* mutation was sufficient to initiate hyperplasia in SOX2^+^ cells, we sought to identify specific KRAS target genes. Comparing esophagi and stomachs in which *Kras* or *Kras*/*p53* were manipulated to controls that did not express *Kras* (false discovery rate (FDR) < 5%), we identified 13 genes that were upregulated. These included *Keratin 17* (*Krt17*), which is a known marker of malignancy (Du et al., 2013). Of note, some of these KRAS target genes encode secreted factors (*Serpine1*, *Il1b*, *Cxcl1*, *Cxcl3*, *Cxcl5* and *Cxcl7*) (Fig. 3C and 3E). Importantly, a large fraction of these genes are associated with SASP (Coppe et al., 2008). These genes were upregulated by oncogenic KRAS rather than by P53 modification (Fig. 3E), recapitulating the different impacts of oncogenic KRAS and P53 deletion.

### SASP-related factors are involved in oncogenic Kras-mediated cellular proliferation

**Figure 4:**
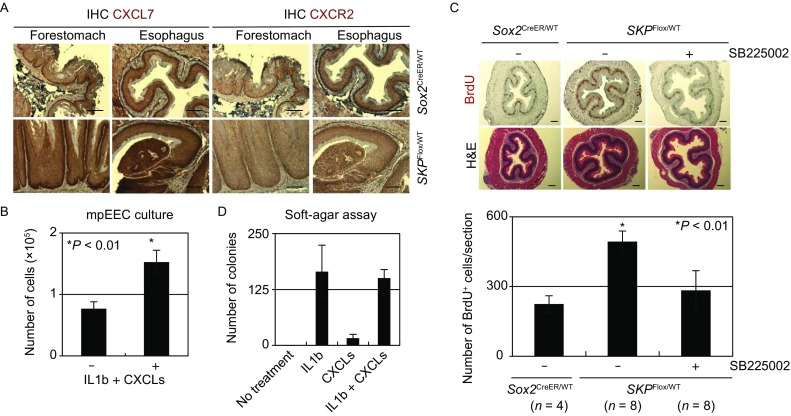
Crucial roles of secretory phenotype on tumor initiation caused by KRAS activation. (A) Expression of CXCL7 and CXCR2 in striated epithelial layers. Scale bars, 100 μm. (B) Effect of chemokines on mouse primary esophageal epithelial cell (mpEEC) proliferation. The isolated esophageal cells were treated with or without recombinant IL1b and CXC ligands (25 ng/mL) for 6 days and then the number of cells was counted. CXCLs: CXCL1, CXCL3, CXCL5 and CXCL7. Data represent the mean with SD (*n* = 3). *P < 0.01; Student’s *t*-test. (C) Top, effect of CXCR2 inhibitor on esophageal cell proliferation of SKP mice treated with TAM for 1 week. IHC for BrdU was performed on the section from the indicated mice i.p. injected with or without SB225002 daily in parallel to TAM administration. Scale bars, 100 μm. Bottom, quantification of BrdU^+^ cells. *n* = the number of sections from *Sox2*^CreER/WT^ mice and two mice for SKP^Flox/WT^ mice. Data represents the mean with SE. ANOVA and Dunnett’s post-hoc test were applied; *P < 0.01. (D) Soft-agar assay using human primary esophageal epithelial cells treated with recombinant IL1b and CXC ligands (25 ng/mL). Cxcls: Cxcl1, Cxcl3, Cxcl5 and Cxcl7

Previous reports have indicated that SASP accelerates the proliferation of tumor cells while inhibiting the proliferation of surrounding wild-type cells (Acosta et al., 2008; Coppe et al., 2008; Kuilman et al., 2008). Therefore we first asked if the CXC chemokines pathway is activated in foregut epithelia. As shown in Fig. 4A, CXCL7, encoded by *Cxcl7*, which is one of the upregulated SASP-regulated genes, and CXCR2, which is a receptor for the CXC family of chemokines, are expressed in stratified epithelia of the esophagus and forestomach. This led us to examine the effect of CXC chemokines on cell proliferation. For mouse primary esophageal epithelial cells (mpEECs), chemokine treatment accelerated proliferation, highlighting the involvement of these factors in hyperplasia (Fig. 4B). More importantly, chemical inhibition of the CXCR2 signaling pathway with the compound SB225002 (White et al., 1998) in SKP^Flox/WT^ mice (1-week following TAM exposure) resulted in a marked decrease in proliferating cells (BrdU^+^ cells) and in a thinner hyperplastic layer, to levels comparable to the control mice (Figs. 4C and S9). Analyses of RNA-Seq data from ESCC samples available in public datasets (Tong et al., 2012) showed upregulation of CXC ligands and IL1b (Fig. S10). The ability of CXC ligands and IL1b to enhance tumor effects was also observed in a soft-agar assay utilizing human primary esophageal epithelial cells (Fig. 4D). We next tested CXCR2 inhibitor on human esophageal cell lines: human primary esophageal epithelial cells (hpEECs); non-neoplastic, immortalized esophageal epithelial cells (Het-1A); and ESCC line (OE21). We noticed that CXCR2 inhibitor negatively affected esophageal cell proliferation while not affecting human dermis skin fibroblast (HDF) (Fig. S11), highlighting the importance of CXCR2 in ESCC, consistent with previous report (Wang et al., 2006). Collectively, these data indicate that SASP-related factors play crucial roles in tumorigenesis caused by oncogenic KRAS.

### *p53* deletion results in an invasive phenotype

**Figure 5:**
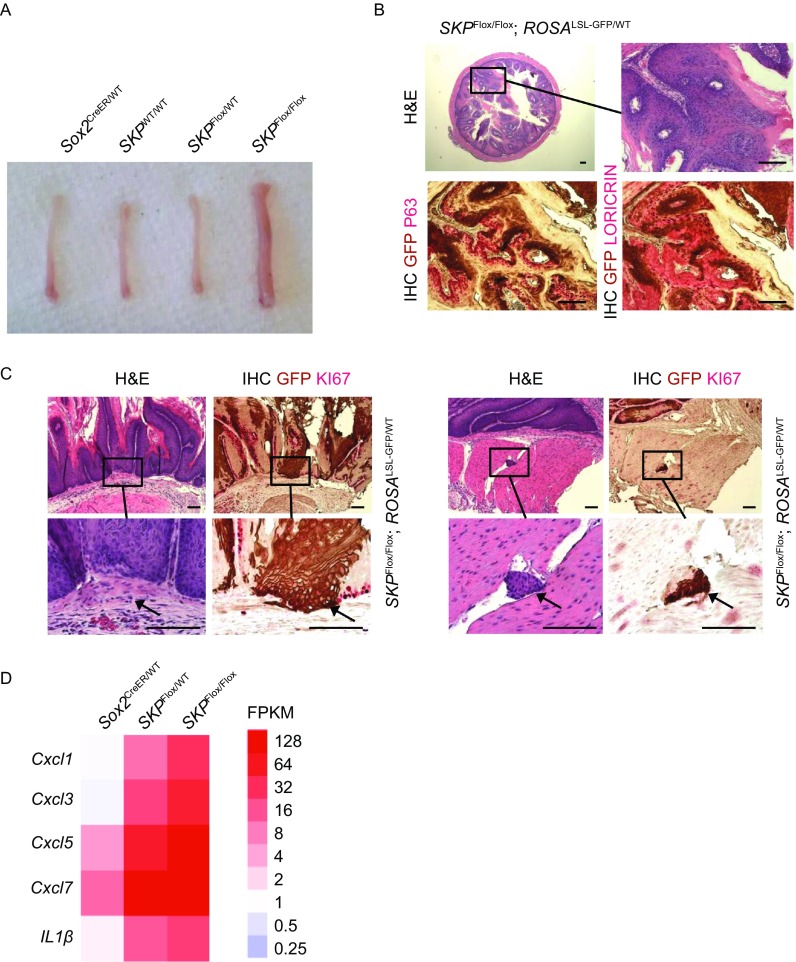
Effect of *p53* deletion on tumor progression. (A) Esophagi from different mouse models. (B and C) Lineage tracing experiment for esophagus (B) and forestomach (C) of SOX2^+^ cells from *SKP*^Flox/Flox^; *ROSA*^LSL-GFP/WT^ mice. The black arrow in Figure 5C shows invasive GFP^+^ tumor cells. GFP^+^ tumor cells, identified by a black arrow, were also observed in normal tissues on the right. Scale bars, 100 μm. (D) Effect of *p53* deletion on expression of SASP-related factors. Heap map of gene expression of SASP-related chemokines described in Fig. 3E

The observation that a *p53* heterozygous background potentiated *Kras*^*G12D*^-induced hyperplastic proliferation led us to further explore the impact of homozygous *p53* deletion on tumor progression. We therefore generated *Sox2*^CreER/WT^; *Kras*^LSL-G12D/WT^; *p53*^Flox/Flox^ (SKP^Flox/Flox^) mice and treated them with TAM for 1 week. Almost all SKP^Flox/Flox^ mice (7 of 8 TAM-treated mice) died within 2 weeks of TAM treatment. This is in contrast to SKP^Flox/WT^ mice, which generally survived 4 weeks. SKP^Flox/Flox^ mice that died following TAM treatment had a much larger esophagus than those of any other genotypes, including SKP^Flox/WT^ mice (Fig. 5A and 5B). It is worth noting that invasion of GFP^+^ cells was only observed in the forestomach of SKP^Flox/Flox^ mice but not SKP^Flox/WT^ mice (Fig. 5C). A higher abundance of SASP-related factors might account for the invasive phenotype (Figs. 5D and S11), in agreement with a previous report (Coppe et al., 2008). Taken together, these results indicate that *p53* homozygous deletion is required for the acquisition of an invasive phenotype.

## Discussion

ASCs/PCs are found in many tissues and organs in the adult body and are important for tissue homeostasis and regeneration upon injury but, at the same time, these cells might be ideal candidates to be the cells-of-origin for cancers (Arnold et al., 2011). Here we found that SOX2^+^ foregut ASCs/PCs are prone to oncogenic transformation despite the presence of SOX2^+^ cells in other organs, such as the lungs. Our observations indicate distinct roles for oncogenic KRAS mutation and P53 deletion in tumor formation. Global gene expression analysis reveals that secreting factors contribute to the development of oncogenic KRAS-induced tumors and highlights a crucial role for the CXCR2 pathway in driving tumor formation.

SOX2 has been reported to play an important role not only in development and somatic reprogramming but also in cancer initiation/progression. For example, amplification of the *SOX2* gene has been reported in human squamous cell carcinomas (SCC) of the lung and esophagus, small-cell lung cancer (SCLC) and glioblastoma (Bass et al., 2009; Annovazzi et al., 2011; Rudin et al., 2012). Overexpression of *Sox2* leads to hyperplasia and tumor formation in several tissues (Lu et al., 2010; Liu et al., 2013; Mukhopadhyay et al., 2014). Furthermore, *Sox2* expression marks the tumor-initiating cell population of skin squamous cell carcinomas once *Sox2* expression is induced during tumorigenesis (Boumahdi et al., 2014). SOX2^+^ cells are also responsible for propagating medulloblastoma and targeting them prevented tumor growth (Vanner et al., 2014). Taken together, these results indicate the importance of the SOX2 molecule and SOX2^+^ cells in tumor development. However, tumor susceptibility of SOX2^+^ cells seems oncogene-specific. A previous report showed that the loss of APC in pyloric SOX2^+^ cells generated tumors (Sarkar et al., 2016). Similarly, targeted expression of oncogenic β-catenin in SOX2^+^ cells is reported to give rise to other tumor types in a non-cell-autonomous manner (e.g., pituitary tumors) (Andoniadou et al., 2013). However, we did not observe abnormal proliferation in the glandular region as well as in the pituitary in our system. These results suggest distinct oncogenic mutation susceptibilities in SOX2^+^ cells throughout different tissue niches.

We also found distinct roles for KRAS and P53 in oncogenic transformation of SOX2^+^ cells. Oncogenic *Kras* expression, but not *p53* deletion, was sufficient to induce a hyperplasic phenotype; and *p53* deletion accelerated tumorigenic proliferation in *Kras*^*G12D*^-induced hyperplasia. Similarly, others have found that the loss of *p53* in stem cells of the colon results in tumor formation only when combined with DNA damage and chronic inflammation (Schwitalla et al., 2013; Davidson et al., 2015). Importantly, *p53* homozygous deletion along with the *Kras* mutation led to an invasive phenotype and highly malignant tumors, highlighting the role of P53 in tumor invasion.

We identified SASP-related chemokines as responsible factors for oncogenic *Kras*-dependent proliferation in the forestomach and esophagus. It is thought that SASP may be induced in senescent cells to potentiate cell proliferation of surrounding pre-tumor cells and to functionally disrupt normal tissues (Krtolica et al., 2001; Coppe et al., 2008). Some of the SASP-related chemokines activate the CXCR2-dependent signaling pathway, known to trigger a secretory network that results in growth arrest, further preventing tumor progression (Acosta et al., 2008). In fact, a previous paper showed that CXCR2 is a blockade to drive oncogene-induced senescence in pancreatic tumors (Lesina et al., 2016). Inconsistent with these reports, we found that oncogenic KRAS increased the expression of SASP-related chemokines in foregut basal cells, which contributed to oncogenic proliferation. Given that epithelial cells in the esophagus and forestomach are highly proliferative, similar to pre-tumor cells, these cells might have unique characteristics, which allow them to proliferate in response to SASP-related chemokines. Interestingly, esophageal epithelial cells express some of the pluripotency factors (unpublished data), highlighting the uniqueness of these cells.

The array of genetic tumor models generated, combined with the lineage tracing experiments and global expression analyses described here, may open new paths for a better understanding of neoplasia. They may also help the future design of therapeutics targeting the initial stages of tumor formation and progression as well as facilitate the identification of novel parameters for earlier tumor diagnosis.

## Materials and Methods

### Mice

*Mcm2*^CreER/WT^ (Pruitt et al., 2007), *Sox2*^CreER/WT^ (Arnold et al., 2011), *Lgr5*^CreER/WT^ (Barker et al., 2007), *Kras*^LSL-G12D/WT^ (Jackson et al., 2001), *p53*^Flox/Flox^ (Jonkers et al., 2001), *ROSA*^LSL-PIK3CA(H1047R)/LSL-PIK3CA(H1047R)^ (Adams et al., 2011), *ROSA*^LSL-Luc/LSL-Luc^ (Safran et al., 2003), and *ROSA*^LSL-GFP/LSL-GFP^ (Mao et al., 2001) have been previously described. We used both male and female mice for this study but the same gender was used for each experiment unless otherwise stated.

To activate CRE in the mice carrying CreER, TAM, dissolved in corn oil, was given orally (50 mg/mL) or intraperitoneally (20 mg/mL) to 6- to 10-week-old animals for 5 consecutive days, unless otherwise stated.

### Tissue preparation and IHC

For IHC, tissues were harvested, fixed in 10% neutralized Formalin for 2 days and then stored in 70% ethanol until further processing. H&E staining, PAS staining and IHC on paraffin-sections were performed following standard protocols. The following antibodies were used for IHC: anti-GFP (Abcam, 6673, 1:200; Clontech, JL-8, 1:100); Ki67 (Cell signaling, 12202, 1:200); Proton-pump (MBL, D032-3H, 1:100); Gastrin (Santa Cruz, sc-783, 1:200); anti-p63 (Santa Cruz, sc-56188, 1:200); anti-CK13 (Abcam, 92551, 1:1000); anti-Loricrin (Abcam, 24722, 1:1000); anti-CXCL7 (Bioss Inc., A-21235, 1:200); anti-CXCR2 (Abcam, 14935, 1:200).

### IVIS experiment

Mice were examined at 3 or 4 weeks post TAM administration by BLI performed using an IVIS Kinetic 2200 from Caliper Life sciences. Mice were i.p. injected with 150 mg/kg D-Luciferin (BIOSYNTH), anesthetized with isoflurane and dorsal images were then captured 10 min post luciferin injection.

### RNA-sequence

Isolated tissues were homogenized with a polytron in TRIzol. The extracted RNA was purified using the RNeasy Micro Kit (Qiagen) from the homogenates. RNA quality was assessed and all samples had a minimum RNA integrity number (RIN) of 7.8. RNA library preps were prepared using the Illumina TruSeq Stranded Total RNA Sample Prep kit with Ribo-zero Gold (cat. no. RS-122-2301). Briefly, RNA was depleted of ribosomal RNA and mitochondrial RNA, then fragmented and reverse transcribed. cDNA was end-repaired, adenylated, ligated with sequencing primers and PCR amplified. Libraries were pooled and sequenced on the HiSeq 2500 using v4 sequencing reagents at single-end 50 base-pair (bp) to a depth of 15–20 million reads per experiment. Reads were mapped to the mouse genome (NCBI37/mm9) using STAR (PMID: 23104886). Gene expression levels and Gene Ontology enrichment were calculated using HOMER (PMID: 20513432) and clustering was performed using Cluster 3.0 and Java TreeView. Differential expression was defined using a false discovery rate (FDR) cut-off of 5% and a fold change of at least 2 using edgeR (PMID: 19910308). RNA-Seq data have been deposited in the Gene Expression Omnibus under accession code GSE66457.

### BrdU labeling

BrdU labeling was performed using BrdU In-Situ Detection Kit (BD Biosciences, 550803) according to the manufacturer’s instructions. Briefly, the mice were i.p. injected with 1 mg of BrdU and the tissues were collected from the injected mice at 24 hr post injection, followed by paraffin embedding and sectioning. After being deparaffinized and antigen-retrieved, the section was stained using biotinylated anti-BrdU and Streptavidin HRP together with DAB substrate and BrdU^+^ cells were counted for quantification.

### Cell culture

Mouse primary esophageal cells were derived as previously described (Kalabis et al., 2008). Briefly, the esophagi were isolated, opened longitudinally, washed in PBS followed by Dispase (1 U/mL) for 15–20 min at 37 °C. The opened esophagi were minced with forceps and incubated with TrypLE for 10 min at 37 °C. After inactivation of TrypLE with FBS, the cell suspension was filtered through 100-μm and 40-μm cell strainers. The obtained cells were centrifuged and re-suspended in keratinocyte serum-free medium (Life Technologies), followed by plating on matrigel-coated plates. Human primary esophageal epithelial cells were obtained from Cell Biologics. Het-1A cell line was obtained from ATCC. OE21 cell line was obtained from sigma. The cells were cultured according to manufacturer’s instructions.

### FACS analysis

Single cell suspension of the esophagus and the forestomach was obtained as mentioned above. Lung cell isolation was performed as previously described (Gereke et al., 2012). Briefly, lungs were perfused with PBS and the salivary glands were removed to expose the trachea, followed by instillation with 1 U/mL dispase and 1% low-melting agarose. After gel solidification with ice, the lungs were isolated and washed with PBS, and incubated with dispase at room temperature for 45 min. The lungs were minced and filtered through 100-μm and 40-μm cell strainers to obtain a single cell suspension. The single cell suspension was subjected to FACS analysis.

### Soft-agar assay

The cells of interest were cultured in 0.5% soft agarose layered on harder agarose in 60-mm dishes. After 14 days, the colonies were counted.

## Electronic supplementary material

Below is the link to the electronic supplementary material.

Supplementary material 1 (PDF 1769 kb)
