# Supplementary material for: Mutations in foregut SOX2+ cells induce efficient proliferation via CXCR2 pathway
Source: Protein Cell. 2019 Apr 30;10(7):485–95. doi: 10.1007/s13238-019-0630-3 (PMC6588654; doi:10.1007/s13238-019-0630-3)
Supplement: Supplementary file 1 — Supplementary material 1 (PDF 1769 kb) [file 13238_2019_630_MOESM1_ESM.pdf]

Supplementary Figure S1. BLI analysis on the lineage-traced cells in response to oncogenic KRAS.

(A) BLI analysis of MKP, SKP and LKP mice. BLI analysis of whole mice (left) and specific tissues (right) 3 weeks post TAM administration to the indicated mice. Li: Liver; H: Heart; E: Esophagus; St: Stomach; Du: Duodenum; SI: Small intestine; C; Colon; Sp: Spleen; Lu: Lung; K: Kidney; P: Pancreas; B: Brain.

(B) BLI analysis of the mice carrying *p53*$^{\mathrm{Flox/WT}}$; *Kras*$^{\mathrm{LSL\text{-}G12D/WT}}$; *ROSA*$^{\mathrm{LSL\text{-}Luc/WT}}$ in the presence or absence of Sox2-CreER. Li: Liver; H: Heart; E: Esophagus; St: Stomach; Du: Duodenum; SI: Small intestine; C; Colon; Sp: Spleen; Lu: Lung; K: Kidney; B: Brain.

Supplementary Figure S2. Histology of the SOX2-expressing tissues of SKP$^{\mathrm{Flox/WT}}$ mice.

No tumor formation was observed in any tissues other than the esophagus and stomach in Fig. 2C.

Supplementary Figure S3. *Sox2*-GFP expression in esophagus, stomach and lung tissues.

FACS analyses of *Sox2*$^{\mathrm{GFP/WT}}$ and wild-type littermate controls show GFP$^+$ cells in the indicated tissues.

Supplementary Figure S4. GFP-based lineage tracing experiment.

FACS analyses of *ROSA*-GFP$^+$ cells in the lineage tracing experiment. The indicated mice were treated with TAM and the esophagus and forestomach were collected 1-week post TAM administration. The obtained cell-suspension after digestion was subjected to FACS analysis. The mice carrying *ROSA*$^{\mathrm{LSL\text{-}Luc/WT}}$ were used as a control for FACS analyses.

Supplementary Figure S5. Alterations in the glandular stomach in SKP$^{\mathrm{Flox/WT}}$ mice.

(A) H&E and Periodic acid-Schiff (PAS) staining of glandular stomachs collected from SKP$^{\mathrm{Flox/WT}}$ 3 weeks post TAM administration. Scale bars, 100 μm.

(B) Co-staining for GFP and differentiated markers (PROTON-PUMP and GASTRIN) on lineage tracing samples. Scale bars, 100 μm.

Supplementary Figure S6. Lack of effect of oncogenic *Kras* on the differentiation potential of SOX2$^+$ cells.

IHC for undifferentiated (P63) and differentiated (CK13 and LORICRIN) markers on paraffin-embedded sections from SKP mice. Scale bars, 100 μm.

Supplementary Figure S7. Hyperplasia from SOX2$^+$ cells with activation of PI3K pathway.

(A) Schematic representation of genetic makeup for tumor initiation models for *PIK3CA*$^{\mathrm{H1047R}}$.

(B) Hyperplasia in esophagus of *Sox2*$^{\mathrm{CreER/WT}}$; *p53*$^{\mathrm{Flox/WT}}$; *ROSA*$^{\mathrm{LSL\text{-}H1047R/LSL\text{-}GFP}}$ mice after TAM administration.

(C) Lineage tracing experiment of SOX2$^+$ cells from *Sox2*$^{\mathrm{CreER/WT}}$; *p53*$^{\mathrm{Flox/WT}}$; *ROSA*$^{\mathrm{LSL\text{-}H1047R/LSL\text{-}GFP}}$ mice post 4-week TAM administration. Co-staining of GFP with P63 or LORICRIN.

Supplementary Figure S8. Effect of p53 deletion on oncogenic *Kras* driven hyperplasia.

Co-staining for GFP and KI67 on lineage tracing sample from the indicated mice

1-week post TAM administration. Scale bars, 100 μm.

Supplementary Figure S9. BrdU labeling for forestomach from SKP mice treated with or without CXCR2 inhibitor.
IHC for BrdU on the section from the stomach of BrdU-injected SKP mice treated with TAM in the presence or absence of SB225002. The number of $BrdU^+$ cells in a bulge was counted on 30 independent fields of the sections from two mice for the indicated condition. Data represent the mean with SE. *P <0.01; Student's t-test. Scale bars, 100 μm.

Supplementary Figure S10. Expression of chemokines in human ESCC samples.
Box plot of indicated chemokines in previously published RNA-Seq data set of human ESCC (Tong et al., 2012).

Supplementary Figure S11. Negative impact of CXCR2 inhibitor on the cell proliferation of human esophageal cell lines.
Each cell line was seeded at $1.25x10^4$ cells to 24-well and was treated with 25 or 50 μg/mL SB225002 (SB) on the next day. Cell number was counted 3 days after seeding. Data represent the mean with SD.

Supplementary Figure S12. Heat map of the genes that are specifically upregulated with homozygous deletion of *p53.*

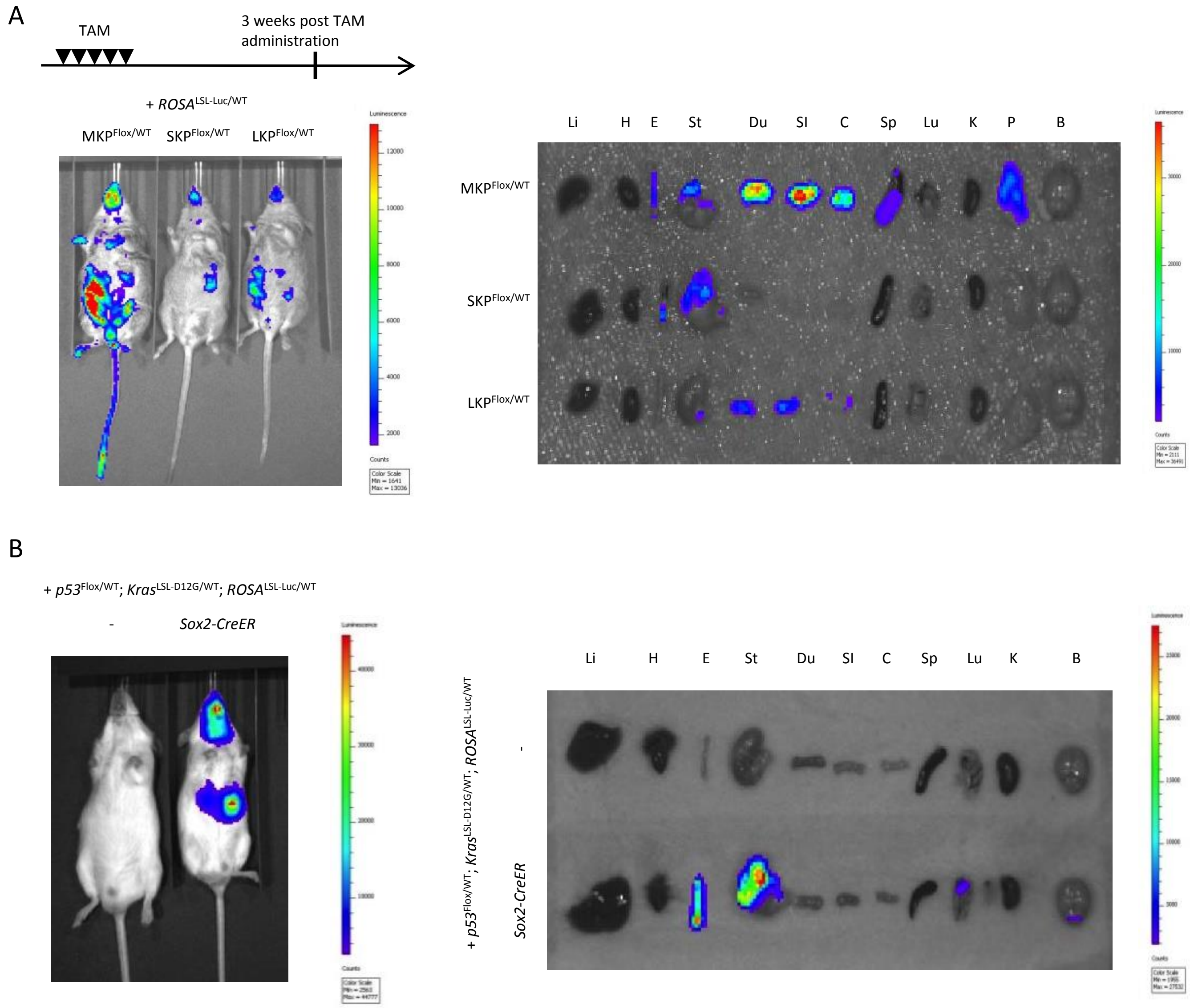

Figure S1, Hishida et al.

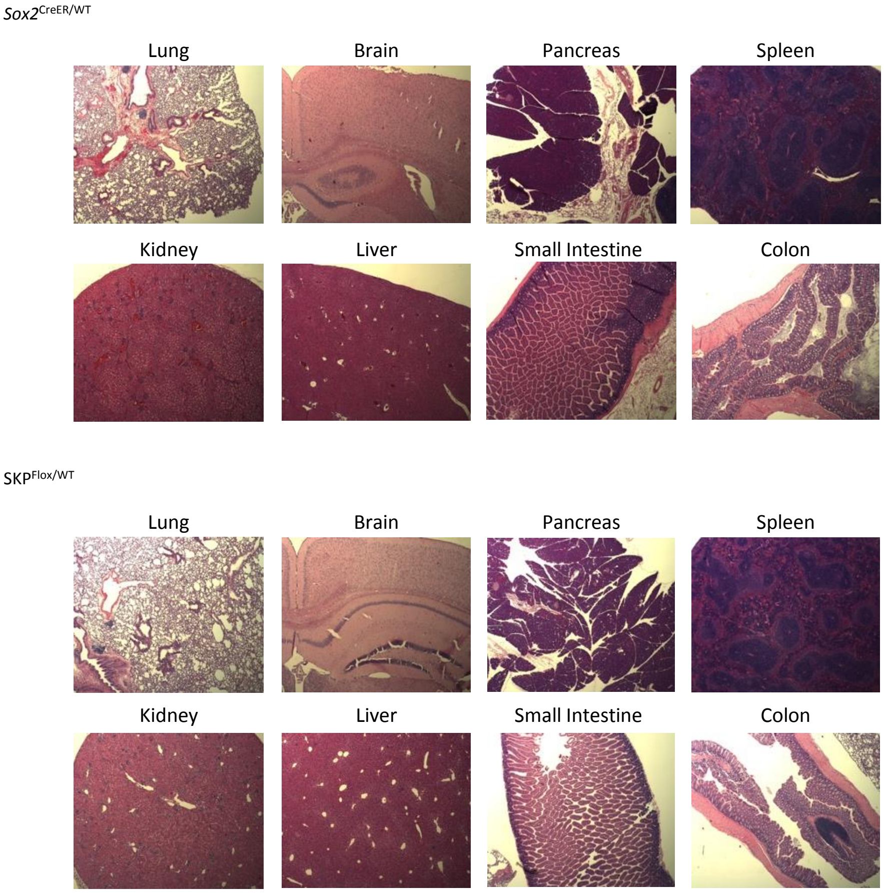

Figure S2, Hishida et al.

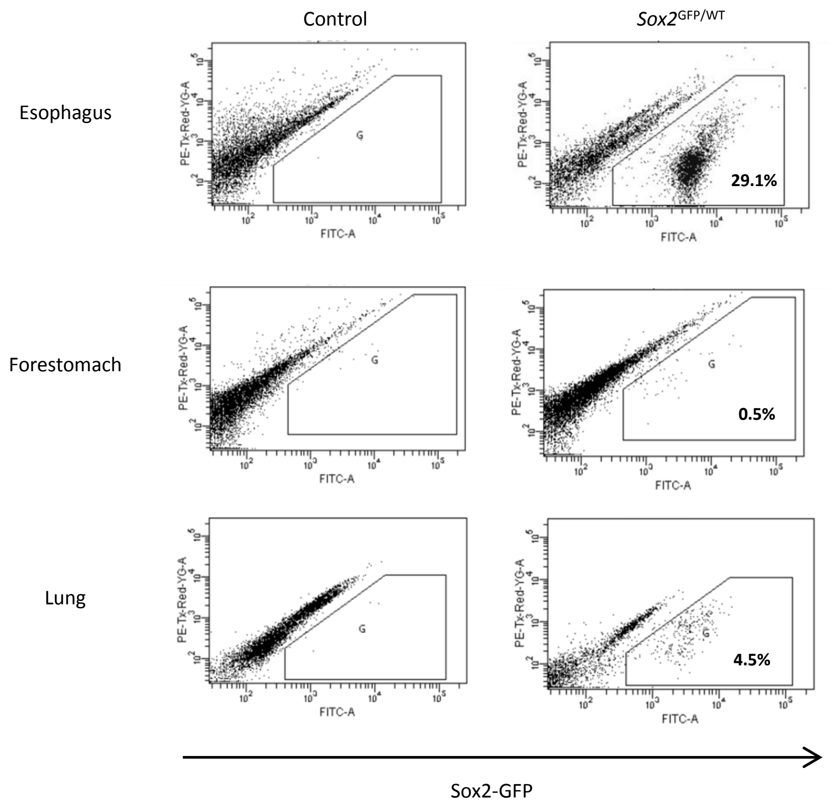

Figure S3, Hishida et al.

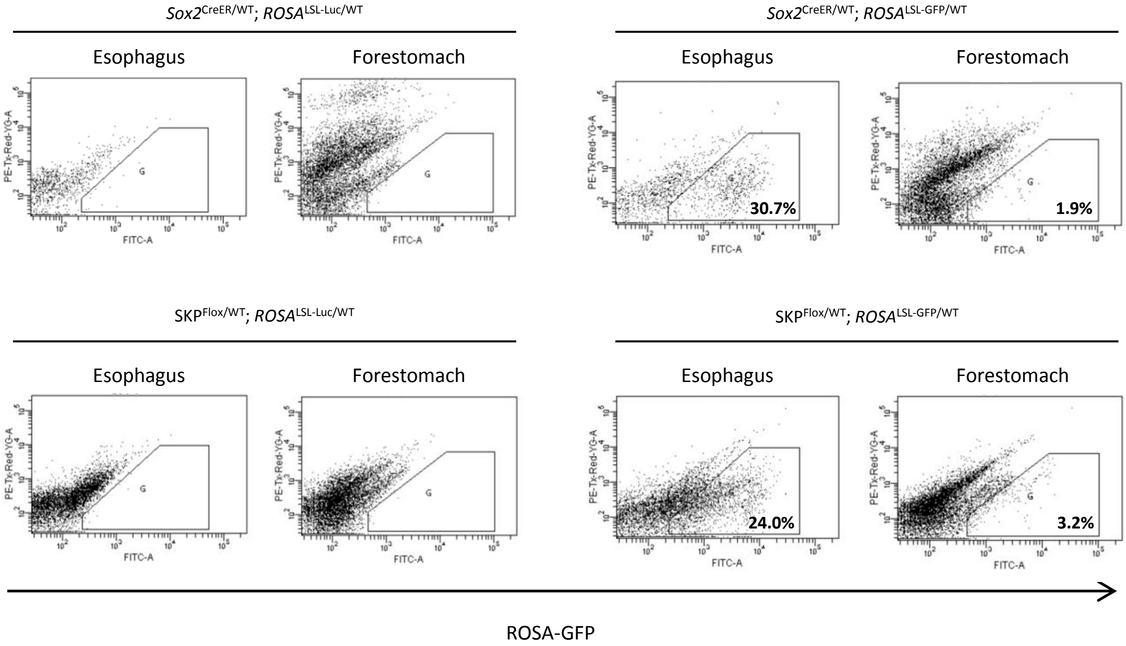

Figure S4, Hishida et al.

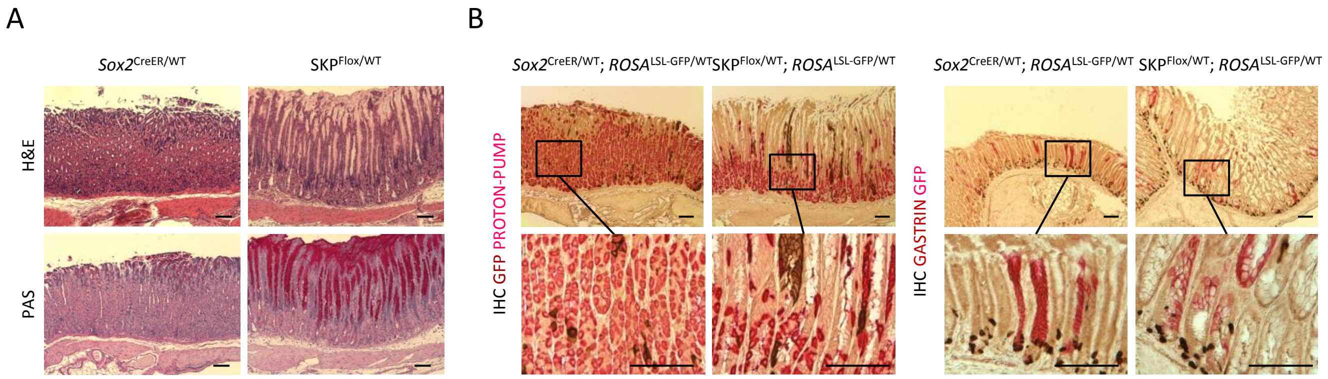

Figure S5, Hishida et al.

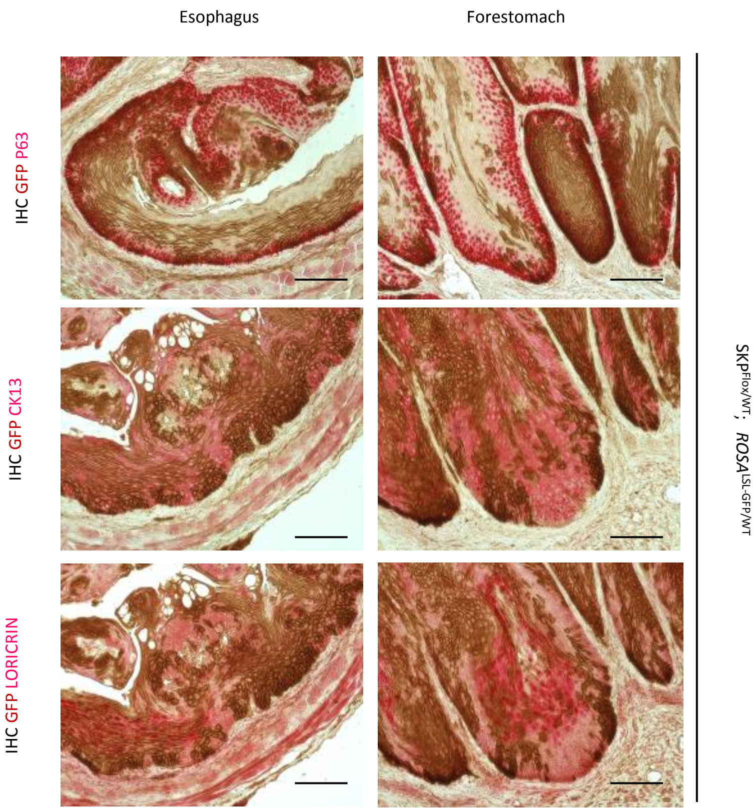

Figure S6, Hishida et al.

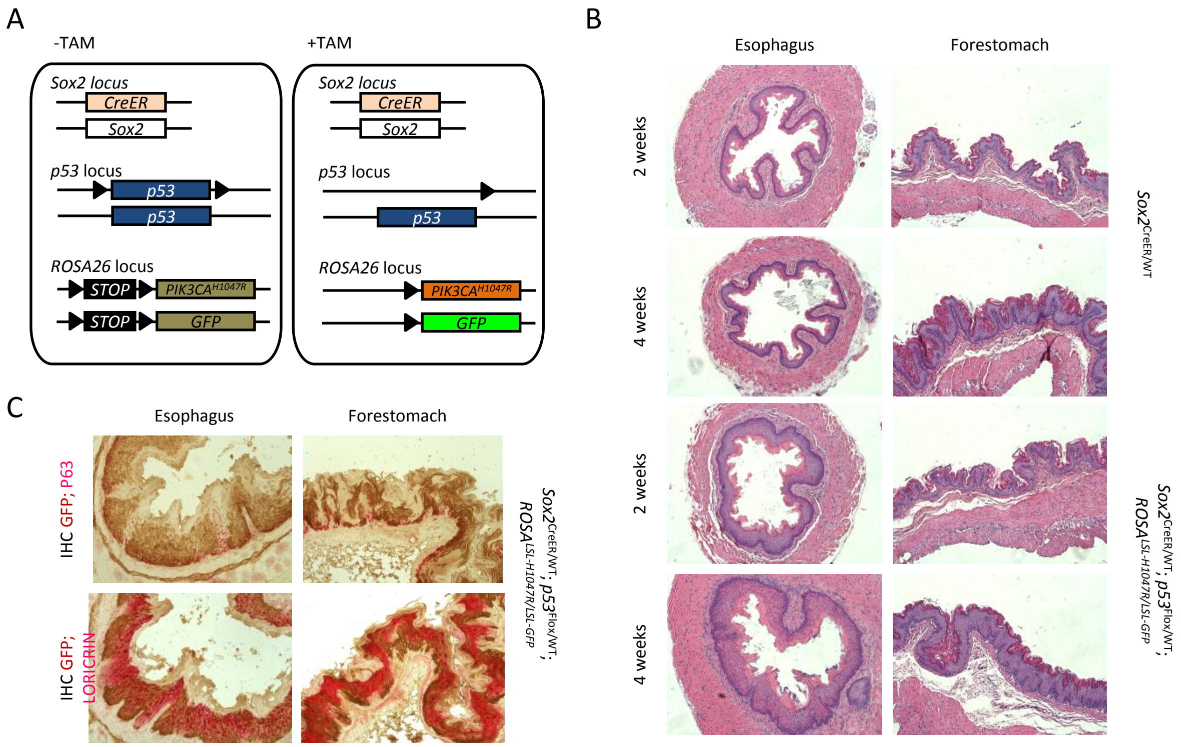

Figure S7, Hishida et al.

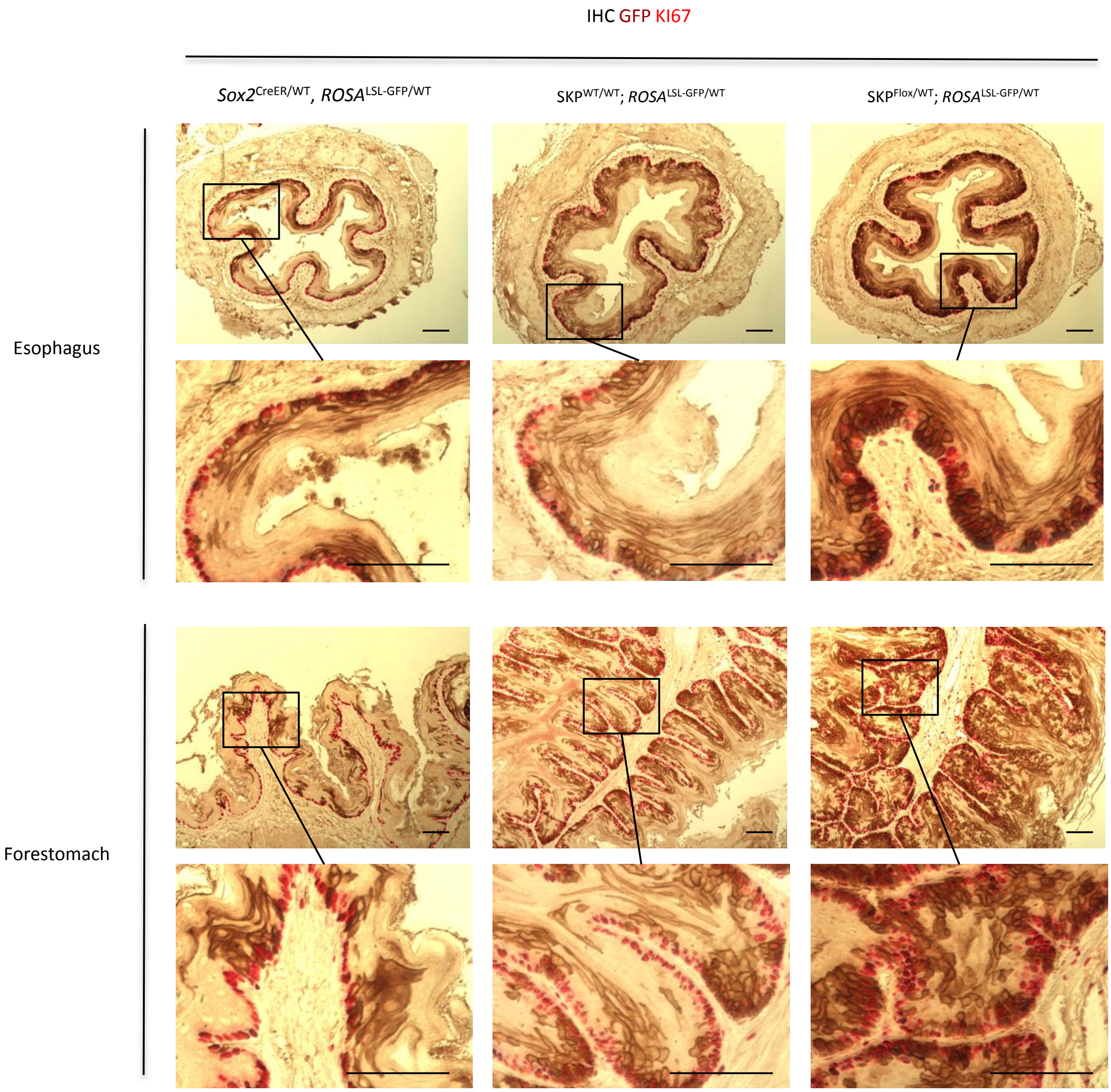

Figure S8, Hishida et al.

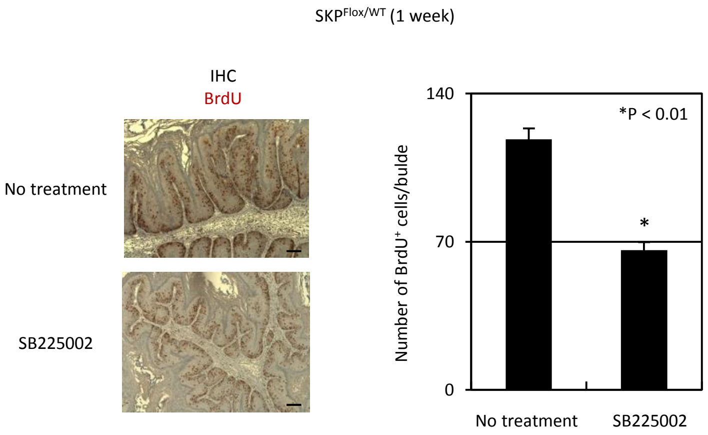

Figure S9, Hishida et al.

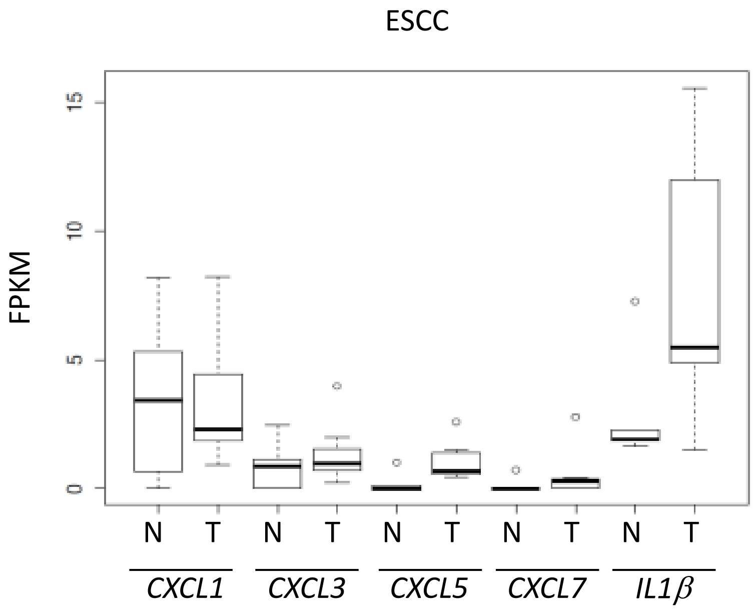

Figure S10, Hishida et al.

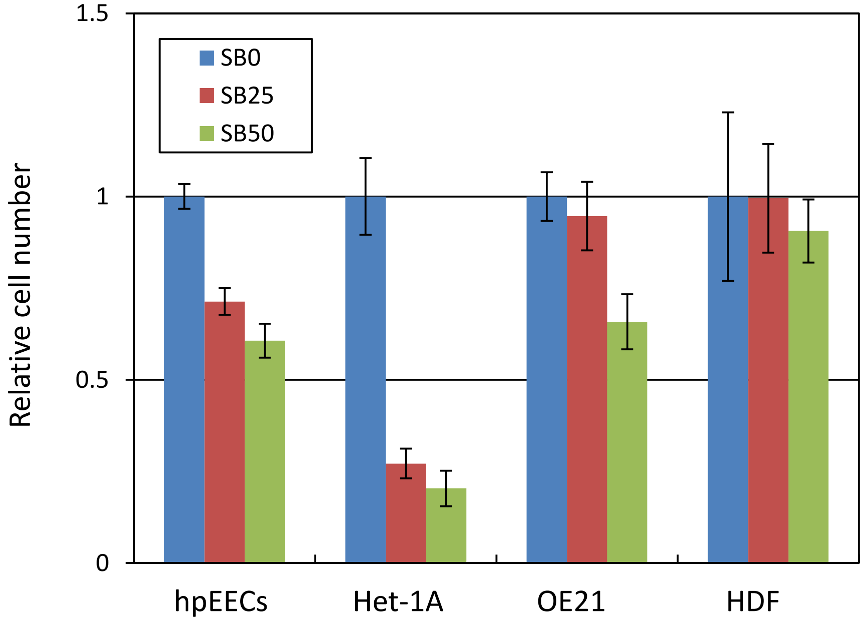

Figure 11, Hishida et al.

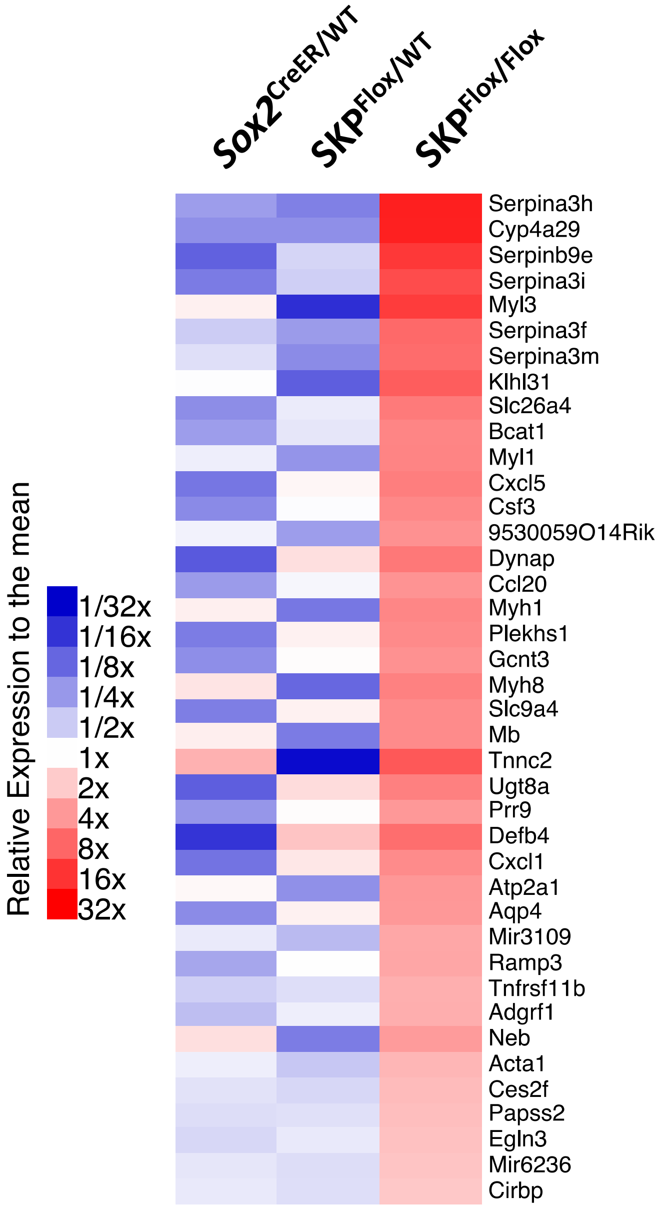

Figure 12, Hishida et al.
